# Still exposed? DDT and PCB levels in Black-headed Gull eggs collected from the Baltic Sea

**DOI:** 10.1007/s10661-026-15987-7

**Published:** 2026-10-07

**Authors:** Magdalena Wlodarz, Friederike Habedank, Angela Schmitz Ornés

**Affiliations:** 1 AG Vogelwarte, Zoological Institute and Museum, University of Greifswald, Greifswald, Germany; 2 State Office for Agriculture, Food Safety and Fisheries of Mecklenburg-West Pomerania, Rostock, Germany

**Keywords:** DDT, PCB, Pesticides, Gulls, Baltic

## Abstract

Legacy pollutants continue to be detected in wildlife and ecosystems, even decades after regulatory action. The study provides the first comprehensive assessment of polychlorinated biphenyls (PCB congeners 28, 52, 101, 138, 153, 180) and chlorinated pesticides, including hexachlorobenzene (HCB), hexachlorocyclohexane (HCH), and dichlorodiphenyltrichloroethane (DDT) and its metabolites in Black-headed Gull (*Chroicocephalus ridibundus*) eggs from the southern Baltic Sea, covering the largest breeding colonies between Germany and Poland. Sampling was conducted over 2 consecutive years. All eggs (*n *= 60) contained DDT and its metabolites and PCB congeners above the reporting level, with mean total PCB concentrations of 0.135–0.268 mg/kg and mean total DDT concentrations of 0.42–0.63 mg/kg. HCB was quantified at low levels (0.01–0.02 mg/kg) in five eggs, which does not allow conclusions about site-specific sources. HCH was not quantified in any sample. No relationships were found between contaminant concentrations and biometric parameters. The consistent occurrence and concentrations of PCBs and DDT metabolites highlight the enduring environmental relevance of these substances. Acute toxic effects were not directly assessed in this study; only associations with measured endpoints were evaluated. Our findings provide crucial new baseline data for the Baltic Sea region and reinforce the value of Black-headed Gull eggs as bioindicators of legacy and chemical exposure.

**Supplementary Information:**

The online version contains supplementary material available at https://doi.org/10.1007/s10661-026-15987-7.

## Introduction

### Persistent organic pollutants: regulatory frameworks, global distribution, and biological consequences

Persistent organic pollutants (POPs), such as polychlorinated biphenyls (PCBs) and organochlorinated pesticides like dichlorodiphenyltrichloroethane (DDT), hexachlorocyclohexane (HCH), or hexachlorobenzene (HCB), are exclusively human-made and highly resistant to degradation with a long history of use in agriculture and industry (Sánchez et al., 1993). These compounds accumulate in aquatic food webs, posing risks to top predators and prompting international regulation on the Stockholm Convention (Dabrowska et al., 2017; Daley et al., 2011; Jayaraj et al., 2016; Zhou et al., 2025). Despite bans in Europe since the 1970s and 1980s, legacy POPs remain detectable in the Baltic Sea ecosystem due to their persistence, long-range transport, and bioaccumulation (Jonsson et al., 2000; Potrykus et al., 2003; Zhou et al., 2025).

Polychlorinated biphenyls (PCBs) are compounds with varying numbers and positions of chlorine atoms on the benzene rings (Beyer & Biziuk, 2009). Introduced in the 1920s for use in electrical equipment and flame retardants, PCBs have been shown to have negative effects on reproduction and development in animals (Fliedner et al., 2012).

The organochlorine insecticide DDT was widely used in agriculture during the mid-twentieth century. The parent compound DDT breaks down into the metabolites dichlorodiphenyldichloroethylene (DDD) and dichlorodiphenyltrichloroethane (DDE), depending on environmental conditions (Ricking & Schwarzbauer, 2012; Zhou et al., 2025). The primary isomers, p,p′-DDT and o,p′-DDT, maintain their chlorination pattern during this transformation. Under anaerobic conditions, DDT mostly turns into DDD, while under aerobic conditions, it turns into DDE (Ricking & Schwarzbauer, 2012). Both DDE and DDD are highly persistent and build up in lipid-rich tissue of living organisms through the food chain (Ricking & Schwarzbauer, 2012; Zhou et al., 2025). While DDT is still used in some tropical countries for vector control, its use has been banned in Germany and the rest of the European Union since the 1970s (Jayaraj et al., 2016; Padayachee et al., 2023).

Hexachlorobenzene (HCB) was once widely used as a fungicide and pesticide, but it is now recognized as a persistent pollutant. In addition to its intentional use, HCB is also produced unintentionally as a byproduct of combustion and industrial processes. Hexachlorocyclohexane (HCH) refers to a group of chemicals with several isomers, including α-HCH, β-HCH, and γ-HCH (Lindane), which was widely used as an insecticide (Kumari et al., 2024). Both compounds have a history of agricultural use but were banned in the 1980s due to their health and environmental impact.

### Relevance of PCBs and chlorinated pesticides for seabirds

A notable case of DDT contamination affecting bird populations was documented by the British researcher Ratcliffe in the 1950s. Ratcliffe (1967) showed that DDT exposure led to eggshell thinning in peregrine falcons (*Falco peregrinus*) and sparrowhawks (*Accipiter nisus*), with similar effects later confirmed in 14 other bird species (Hickey & Anderson, 1968; Lundholm, 1997). The main findings indicate that DDT reduces calcium in eggs, causing thinner shells and lower hatching success (Lundholm, 1997). Seabirds are highlighted as especially at risk, as they accumulate high concentrations of DDT from their fish-based diets, reflecting bioaccumulation in top predators (Kuo et al., 2022; Lundholm, 1997). Laboratory tests with mallards further demonstrated reproductive failure and increased embryo mortality from DDT (Heath et al., 1969). Similarly, lipophilic compounds such as HCB, HCH, and PCBs accumulate in egg yolks of birds at higher trophic levels (Beyer & Biziuk, 2009).

Building on these findings, egg-based studies are widely used because eggs provide a non-invasive tool for pollution monitoring (Pusch et al., 2005; Verreault et al., 2006). In addition, chicken eggs intended for human consumption are tested for toxins by food safety authorities (Petrlik et al., 2022; Reindl et al., 2020); thus, existing methods can be easily applied to ecological questions. Unlike humans, birds eliminate certain harmful substances through hepatic and renal excretion, and females also use their eggs for this purpose (Li et al., 2021; Price, 2017). Seabirds, in particular, serve as sentinel species for biomonitoring coastal regions because changes in their habitat directly affect their breeding sites and health (Reindl et al., 2019, 2020). Previous studies from the Baltic Sea region provide information on persistent organic pollutants in soils (Dabrowska et al., 2017; Szlinder-Richert et al., 2012; Zhou et al., 2025), water (Szlinder-Richert et al., 2012), fish (Falandysz et al., 2004; Kannan et al., 2002), and bodies of top predator birds (Fenstad et al., 2016; Reindl et al., 2020) or eggs (Becker et al., 1992; Fliedner et al., 2012; Holz & Starke, 1990; Knudtzon et al., 2021; Thorstensen et al., 2021; Verreault et al., 2007).

This study quantifies residues of DDT and its metabolites, PCB, HCB, and HCH in Black-headed Gull (BHG) eggs collected in 2023 and 2024, evaluating the continued presence and concentrations of these legacy POPs in the ecosystem after years of non-use. In doing so, it builds upon the work of Holz and Starke (1990), who documented high levels of DDT and PCBs in Black-headed Gull eggs from the same region. By comparing current contaminant loads with historical data, we assess the effectiveness of regulatory measures and the ongoing risk posed to local bird populations. Specifically, the present study analysed legacy POPs in Black-headed Gull eggs from the two largest breeding colonies in the Southern Baltic Sea. The objectives of this study were to (1) determine the concentrations of PCB, DDT and its metabolites, HCB and HCH in gull eggs; (2) compare present contaminant levels with historical data of Holz and Starke (1990); and (3) assess potential associations between maternal body condition — as inferred from biometric measurements — and egg characteristics, with eggshell thickness measured as an indirect indicator potentially associated with DDT exposure. The aim is to provide new insights into the persistence of legacy POPs in avian ecosystems and to support ongoing environmental monitoring and risk assessment efforts.

## Methods

### Site-specific sampling and historical comparison

**Fig. 1:**
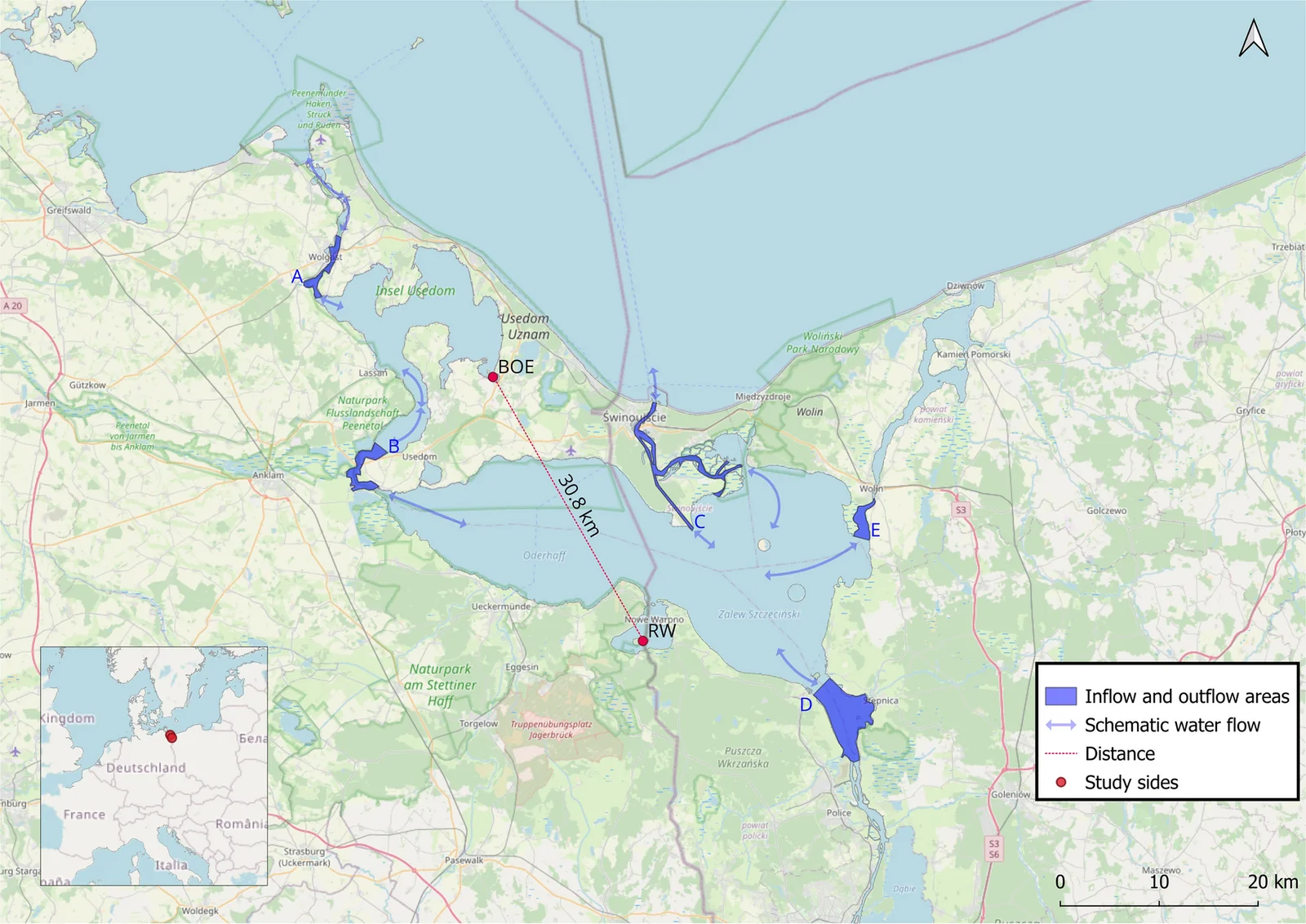
Schematic overview of potential water inflow, outflow, and idealized flow directions at the two study sites (BOE and RW) in lagoon systems along the southern Baltic coast. BOE is positioned in a semi-enclosed sub-basin of the Achterwasser lagoon close to the island of Usedom (A, B), while RW is located in the more exposed Now Warpno Lake, a sub-basin of the Szczecin Lagoon (in Polish: Zalew Szczeciński). The illustrated exchange zones and flow arrows are conceptual, intended to visualize expected differences in water exchange between the two locations based on their geomorphological and hydrological context. Map created using QGIS

This study focused on two major breeding colonies located in the Baltic Region of northern Germany: the Achterwasser Lagoon (including the island of Boehmke, ‘BOE’) and the Szczecin Lagoon (including Riether Werder, ‘RW’) at the German-Polish border (Herrmann et al., 2022). The Oder River, the main freshwater source for the Szczecin Lagoon, delivers high nutrient loads into Europe’s largest lagoon, and sustains water residence times, promoting intense biogeochemical transformations and particle retention. These processes make the lagoon an effective sink for nutrients and pollutants (Friedland et al., 2019; Neumann et al., 2025). In contrast, Boehmke lies in a more sheltered lagoon connected to the Baltic Sea near Usedom (Fig. 1). The Achterwasser Lagoon overall is characterized by stronger water exchange and wind-driven mixing than the river-influenced Szczecin Lagoon; but within it, Boehmke’s sheltered sub-basin may have reduced local water exchange and increased sedimentation relative to the more exposed setting of Riether Werder in the Szczecin Lagoon system. The region is subject to industrial and agricultural runoff, increasing the vulnerability of the Baltic Sea ecosystem (Reckermann et al., 2022). While there is no well-documented source of POPs to the Boehmke area, the Achterwasser system receives diffuse inputs from agriculture, urban drainage around Usedom, and resuspension of contaminated sediments. Studies in southern Baltic have detected PCBs, DDT, and HCB in bottom sediments, with local variability reflecting both historical use and regional/global atmospheric deposition, as well as legacy industrial and agricultural inputs (Dahlberg et al., 2020; Pazikowska-Sapota, 2006).

Riether Werder is influenced by the Oder River catchment, which integrates a larger, mixed land-use watershed with a history of agriculture and industry. Sediments in the broader Szczecin Lagoon contain trace levels of organochlorine pesticides and PCBs, indicating regional legacy contamination, though concentrations were generally lower than in the most polluted hotspots elsewhere (Piotrowski, 2003). Both coastal regions have a history of agricultural POPs use (e.g. DDT until bans in the 1970s–1980s). Sediments act as secondary sources of POPs, releasing contaminants back into water column via diffusion and resuspension, especially where organic-rich sediments occur (Dahlberg et al., 2020).

Over the past decade, both breeding colonies exhibited a stable growth rate (Herrmann, 2017; Herrmann et al., 2022; Ryslavy et al., 2020). During the 2023 and 2024 breeding seasons, nests containing two eggs were tagged, and nests with a third egg were added to the study as they appeared. All eggs were collected following the capture of breeding pairs. The laying order of sampled eggs was not determined. Early-season egg removal enables additional breeding attempts (Makatsch, 1952). From each colony and year, 15 eggs were collected (one random egg per nest; total *n *= 60). Only one random egg was sampled per nest to ensure sample independence and minimize potential bias from laying order. At each nest, both attending adult Black-headed Gulls were captured and measured to allow sex determination by skull length (see the ‘Assessment of female physiological condition and egg characteristics’ section). In total, 47 female BHGs were identified for analysis.

The island of Boehmke was previously included in a study by Holz and Starke (1990), which systematically sampled BHG eggs in 1986 from both coastal and inland breeding colonies to compare contaminant concentrations of DDT and PCBs. Holz and Starke (1990) investigated effects of DDT on eggshells due to unchanged total DDT concentrations since the 1960 s and reports of eggshell thinning in other species, e.g. in Ratcliffe (1967) and Hickey and Anderson (1968), with DDT concentrations being higher in the Baltic Sea than in the North Sea. Compared to other gulls or terns, Black-headed Gulls have exhibited higher contaminant concentrations than other sea bird species (Holz & Starke, 1990). Although these authors provided valuable baseline data on DDT and PCB contamination in the 1980s, up-to-date information on contaminant trends in these colonies is limited, particularly given recent colony growth and persistent environmental pressures.

### Estimation of contaminants

The analytical work was conducted at a recognized official surveillance laboratory in Rostock, Germany. This laboratory is part of the State Office for Agriculture, Food Safety, and Fisheries of Mecklenburg-West Pomerania. It employs an accredited method based on L00.00–34 from the official collection of investigation procedures. This follows Section 64 of the German Foods, Consumer Goods, and Feedstuffs Code (LFGB). The method, as described by Hildmann et al. (2015), has been updated. It aligns with current best practices and has undergone advanced automation and miniaturization.

For analysis, one random egg from each nest was taken, and 0.5 g homogenate (yolk and white, 1:1) was prepared. Each aliquot from the gull egg and a contaminant-free chicken egg (used as a quality control matrix) was transferred in 5-mL PP tubes with steel beads, setting up consistent sample handling. Only fresh, unhatched eggs were collected for analysis. All eggs were collected during the early laying period, prior to the onset of incubation, and were presumed to be fertilized but had not begun development. To prepare the extraction, 50 µL internal standard solution (1 ng/µL, ‘ISTD’, Supplementary Material 1) and approx. 0.5 g sodium sulphate (Na_2_SO_4_) were added with a 3-mL mix of hexane and acetone (v:v 2:1). The quality control sample (5 ng/µL) was spiked with 50 µL of the 0.01 ng/µL pesticide solution and 50 µL ISTD mix (1 ng/mL solution), mirroring sample preparation steps. Each sample was homogenized for 1 min (Homogenisator Beadblaster by Benchmark) and then centrifuged at 4000 rpm for 5 min, facilitating subsequent purification. The supernatants were transferred into 2-mL glass vials and placed in the FREESTYLE system. Further sample preparation on the FREESTYLE automated clean-up system (LC Tech) included modules for gel permeation chromatography (GPC) and programmed evaporation, continuing workflow. 1.5 mL of the supernatant was evaporated to dryness and reconstituted in 5 mL mobile phase for GPC (ethyl acetate:cyclohexane (1:1)). The resulting solution was transferred to the GPC column containing 50 g Biobeads, where chromatographic separation was conducted at 5 mL/min using an elution mixture of ethyl acetate and cyclohexane (1:1). The eluent was collected and again evaporated to dryness and reconstituted in 1.5 mL ethyl acetate.

The final extract was injected into gas chromatographic-mass spectrometry system (GC-MS 7890A-7010 Agilent) for quantification. Target compounds were identified based on retention time, mass transition and the ratio between the quantifier and qualifier (first and second mass transition). The expected intensity ratios were determined from matrix-matched calibration solution measurements, ensuring consistency in analysis. Multiple reaction monitoring (MRM) analyses were performed using established transitions and instrument based on Agilent-supplied method parameters.

Calibration curves were prepared using certified standards over the concentration range of 0.0008–0.1 ng/µL and were generated from the ratios of analyte peak areas to the internal standard peak area. Concentrations in egg samples were calculated from the corresponding calibration equations. The calibration curves showed good linearity (*R*^2^ > 0.99).

The analytical method was validated in an accredited state laboratory for official food control in accordance with SANTE/11312/2021 (European Commission 2026) subject to regular quality assurance, including participation in relevant proficiency testing schemes. Method validation assessed parameters such as linearity, specificity, accuracy (trueness), recovery, precision (expressed as relative standard deviation, RSD), and false-positive/false-negative rates. Matrix-matched quality-control (QC) samples were analysed during initial method validation and ongoing validation. Acceptance criteria for initial validation were 70–120% recovery and < 20% RSD. For routine ongoing QC samples, recoveries between 60 and 140% were considered acceptable. Egg-matrix blank samples were analysed alongside the samples to assess potential background contamination. Blank responses were required to remain below 30% of the lowest validated concentration level. Detailed validation and ongoing QC results are provided in Supplementary Material 2.

For method validation, the lowest concentration level successfully validated in the egg matrix was 0.001 mg/kg, which was considered the limit of quantification (LOQ) according to the laboratory’s SANTE-based approach. A separate method-specific limit of detection (LOD) was not determined. For the present study, however, a more conservative reporting level (RL) of 0.01 mg/kg was applied for the quantitative evaluation and reporting of gull egg samples. Concentrations below this threshold were not considered reliable quantitative results and were treated as zero for statistical analysis. This conservative reporting approach may underestimate true contaminant levels but provides a consistent basis for quantitative evaluation of the study samples.

The study focuses on six non-dioxin-like PCB (ndl-PCB) congeners (PCB 28, PCB 52, PCB 101, PCB 138, PCB 153, and PCB 180). These congeners were part of the routine analytical scope of the method and correspond to the indicator PCBs commonly used in environmental monitoring and assessment. Every PCB congener exhibits different toxicological properties and environmental behaviour, some being more biologically active or more persistent than others. In addition, persistent organochlorine pesticides were analysed, including DDT and its metabolites DDE and DDD, as well as HCH isomers (α-, β-, γ-, and δ-HCH) and HCB. These compounds were selected due to their bioaccumulation potential and relevance as indicators of historical and ongoing contamination.

In this study, the term ‘DDT and its metabolites’ refers to the isomers p,p′-DDT, o,p′-DDT, p,p′-DDE, and p,p′-DDD and o,p′-DDD. HCH isomers (α-HCH, β-HCH, and γ-HCH) were analysed and collectively referred to as HCHs. All concentrations are reported in milligrammes per kilogramme to two decimal places, in accordance with the laboratory’s reporting level of 0.01 mg/kg for all analytes. All values are rounded to match analytical precision. All values are in wet weight (ww), unless stated otherwise.

### Assessment of female physiological condition and egg characteristics

Upon locating a nest, we labelled it with a wooden stick for identification and monitored it for 2 consecutive days. During this observation period, both adult Black-headed Gulls attending the nest were captured using walk-in traps at the nest site. Each adult was ringed for individual identification and measured for skull length using a digital calliper, following the methods of Baker (1993) and Holz and Starke (1984), to distinguish between sexes in adults, as females are generally smaller than males (Indykiewicz et al., 2025). This combination of nest monitoring, individual marking, and biometric measurement allowed us to reliably identify and sample both members of each breeding pair. While skull length is a widely accepted method for sexing Black-headed Gulls, a small degree of overlap exists between sexes, and thus occasional misclassification cannot be completely excluded. Body condition (hereafter body mass index, ‘BMI’) was determined with the following formula: BMI = Body mass (g)/[Skull length (mm)]^*a*^, where *a* is the scaling exponent following Peig and Green (2009) and the modification for gulls by Sebastiano et al. (2021). Body weight can fluctuate throughout the day, and it is influenced by factors such as meal size or timing before capturing the birds (Sebastiano et al., 2023). Therefore, the BMI calculation was used as a more stable variable.

Studies have shown that the thickness of the eggshell can vary depending on the specific measurement location. For example, in Forster’s tern (*Sterna forsteri*) eggs, the blunt pole of the egg had a thinner eggshell on average compared to the sharp pole of the egg (Peterson et al., 2020). To address this variation, we measured eggshell thickness at three positions on each shell: equator (hereafter ‘lateral’), sharp pole, and blunt pole. Eggshells were cut along the equator and, after removing the egg content, rinsed in tap water. The inner membrane was removed manually by mechanical scraping. The selected pieces contained both spotted and pale areas of the eggshell. For each egg, nine measurements (three repetitions per position) were taken without the membrane using an eggshell thickness gauge (model 0.001 mm, Mitutoyo, Sun et al. (2012)). The average measurements for the positions were then used to calculate the mean thickness of the eggshell (hereafter ‘average eggshell thickness’).

Each egg was photographed laterally and from a blunt-pole angle with a Nikon D70s mounted on a wooden box and illuminated with white light to standardize lighting conditions, following Schmitz Ornés et al. (2014). For both positions, camera settings were f/4.5, 1/60 s, and ISO 1600. The images were saved in RAW format to keep data uncompressed for further analysis. Subsequently, egg volume was estimated using the open-source image-analysis toolbox ‘micaToolbox’ in the software ImageJ, in accordance with Troscianko (2014) and Troscianko and Stevens (2019).

### Data processing and analyses

Descriptive statistics (mean, standard deviation, range, detection frequencies) were computed for each contaminant and bird parameter. Visual inspection of distributions and group variances was performed, and data were treated as approximately continuous and independent within groups. HCB and HCH isomers were excluded from further analyses because of their low detection frequency. HCB was detected above the reporting level of 0.01 mg/kg in only five samples, whereas no HCH isomer was detected above this threshold. The analyses were primarily exploratory, aiming to identify potential patterns and effect directions rather than to provide definitive hypothesis testing.

Effects of island and year on total DDT and its metabolites in Black-headed Gull eggs were tested using a two-way linear model with an Island × Year interaction. Island was included as a factor in the two-way linear model to assess potential local differences in contaminant concentrations between the two study sites. The two colonies are situated in distinct lagoon systems (see the ‘Site-specific sampling and historical comparison’ section), which differ in hydrology and potential sources of contamination. Including island as a model factor allowed for examination of site-specific effects alongside inter-annual variation. To address right-skew, we analysed only positive values and modelled log10 (DDT and its metabolites). Model assumptions were assessed via residual diagnostics; robust HC3 standard errors were used as sensitivity checks. Results include Type II ANOVA, *η*^2^, and estimated marginal means (EMMs) that were back transformed to the original scale (mg/kg).

To assess associations between contaminants and eggshell metrics, Spearman’s rank correlation was utilized. Each contaminant (DDT and its metabolites, total PCB) was compared to each eggshell metric (blunt, sharp, lateral, average) to capture monotonic relationships with minimal distributional assumptions. Statistical significance was evaluated at α ≤ 0.05.

All analyses were performed in R (R Core Team, Version 4.4.2) and R Studio (Version 2024.12.0). The following packages were used: ‘dplyr’ (Wickham et al., 2023) to work with data frames, ‘tidyverse’ for workflows (Wickham et al., 2019), ‘broom’ (Robinson et al., 2014) for tidying model outputs, ‘purrr’ (Wickham & Henry, 2025) for modifying data, ‘ggplot2’ for visualizations (Wickham, 2016), ‘viridis’ for colour scales (Garnier et al., 2023), ‘readxl’ for data import (Wickham & Bryan, 2025), ‘kableExtra’ to customize tables (Zhu, 2017), ‘effectsize’ for standardized parameters (Ben-Shachar et al., 2020), ‘car’ for running and diagnosing regression models (Fox & Weisberg, 2024), and ‘ggeffects’ for generating marginal effects and model predictions (Lüdecke, 2017).

The map was produced using QGIS (version 4.0.2). Coastlines and administrative boundaries were obtained from OpenStreetMap (downloaded on 2026-05−01). All spatial data were projected in EPSG:32632 — WGS 84/UTM zone 32 N. Study site polygons and point locations were digitized manually based on high-resolution satellite imagery for visualization purposes. The schematic water inflow and outflow areas, as well as the flow arrows, were added manually for illustrative purposes and do not represent hydrodynamic model output or measured current patterns. The map is intended to provide a conceptual spatial framework for comparing the hydrological settings of the two lagoon systems.

## Results

Among 57 intact eggs, length ranged from 41.8 to 57.4 mm (mean = 52.1 mm, SD = 2.9), width from 33.8 to 40.6 mm (mean = 37.3 mm, SD = 1.33); average egg volume was 36.6 cm^3^ (Supplementary Material 3). All 60 tested eggs contained DDT and its metabolites in addition to PCB congeners above the reporting level. Mean concentrations of PCB, DDT and its metabolites did not differ by year or island (two-way linear model, all *p *> 0.05). Low concentrations of HCB were quantified in five eggs (ranging 0.01 to 0.02 mg/kg), while HCH was not quantified in any sample.

### PCB in Black-headed Gull eggs

**Fig. 2:**
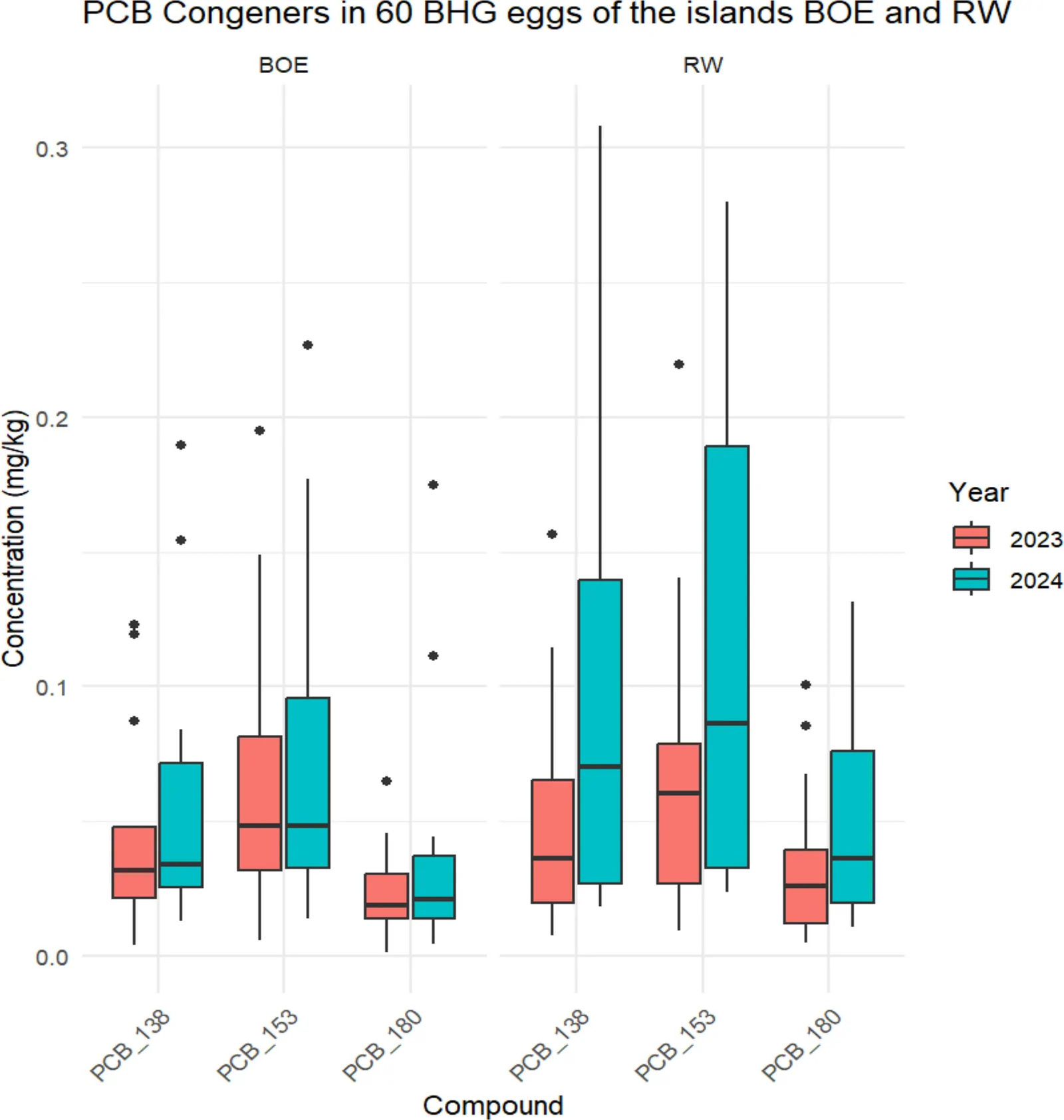
Concentrations of PCB congeners (mg/kg, ww) in Black-headed Gull eggs from Boehmke and Riether Werder (2023–2024). Summary statistics for individual PCB congeners are available in Supplementary Table 4.

The PCB profile was dominated by higher-chlorinated congeners, namely PCB-138, PCB-153, and PCB-180, with mean concentrations above 0.04 mg/kg (median) and 100% detection in all samples (Fig. 2, Supplementary Material 4). Lower-chlorinated congeners (PCB-28, PCB-52, PCB-101) were less consistently quantified and at lower concentrations. The island Riether Werder (‘RW’) showed consistently higher PCB levels than the island Boehmke (‘BOE’), with the highest mean concentration recorded at RW in 2024 (PCB 0.268 mg/kg). The total mean concentration of all measured PCBs was 0.18 mg/kg (*n *= 60), compared to the historical mean total PCB concentration of > 2 mg/kg reported by Holz and Starke (1990) for the island BOE in 1986. Unfortunately, Holz and Starke (1990) did not distinguish between PCB congeners and none of the authors had the original data anymore when asked in 2024.

### DDT, HCH, and HCB in gull eggs

**Fig. 3:**
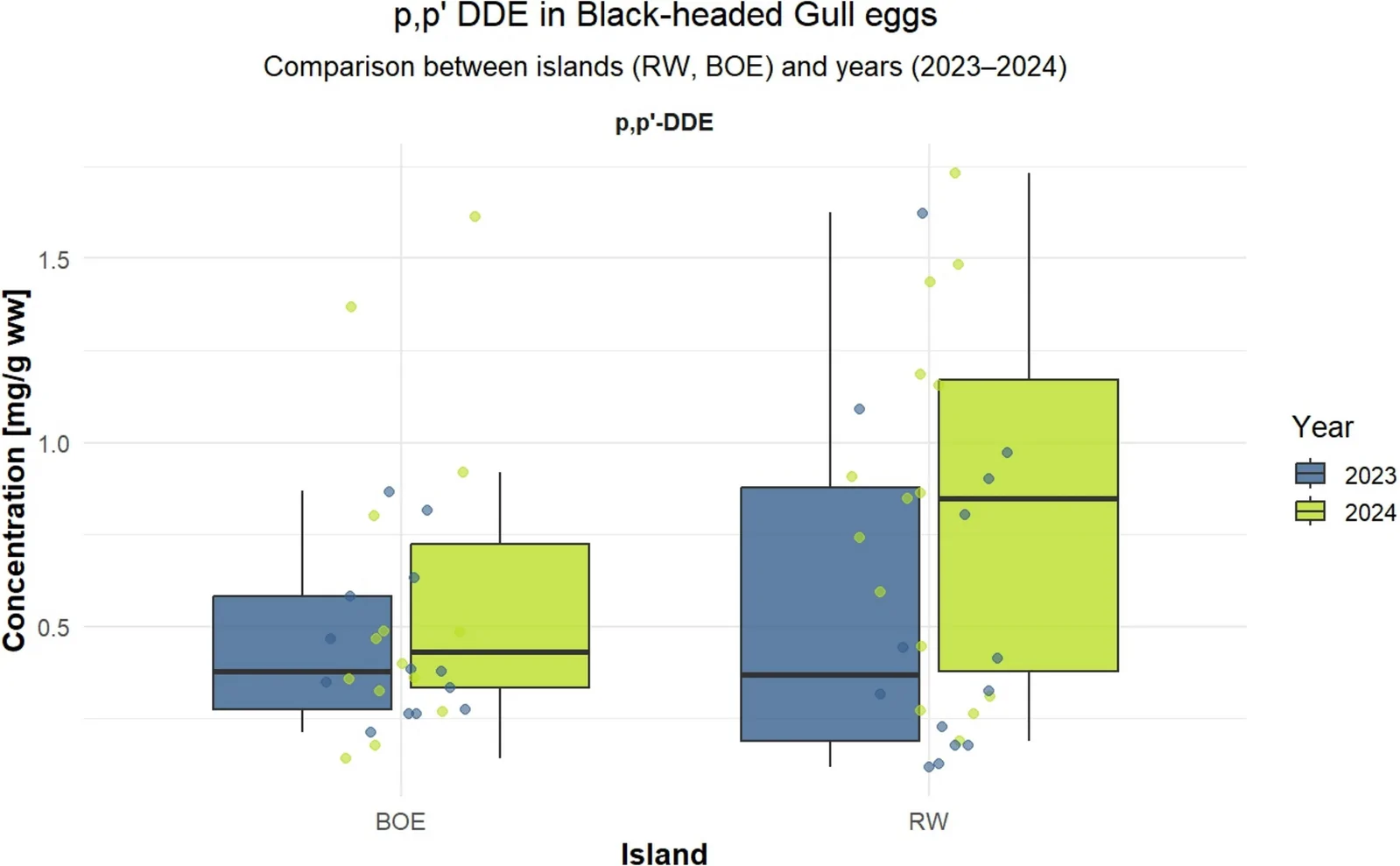
Occurrence of p,p′-DDE across years: Despite universal presence, the DDT metabolite did not differ between 2023 and 2024, supporting a primarily legacy (rather than ongoing) DDT input signal

Among DDT and its metabolites, DDE (o,p′- + p,p′-DDE) was quantified in 100% of egg samples (Table 1, Fig. 3) and was the predominant compound. p,p′-DDE accounted for the majority of the residues (mean = 0.57 mg/kg) while o,p′-DDE was below the reporting level. p,p′-DDT was present in 33 out of 60 eggs (55%) at concentrations up to 0.13 mg/kg. o,p′-DDT was below the reporting level in all samples. Among DDD isomers, only a single sample (island RW, 2023) contained p,p′-DDT at 0.01 mg/kg, while all other DDD isomers were below the reporting level (Table 1).

**Table 1:** Detection frequencies and concentrations of HCH, HCB, and DDT and its metabolites in Black-headed Gull eggs from all years combined (*n *= 60; only values with reporting level ≥ 0.01 mg/kg were included; minimum, maximum, and mean concentrations were calculated based on quantified samples only. Samples in which analyte concentrations were below the reporting level were excluded from these summary statistics). Descriptive statistics for individual PCB congeners are added to Supplementary Material 4

Compounds	*n* detected (absolute)	% detected	Minimum (mg/kg)	Maximum (mg/kg)	Mean (mg/kg)
p,p′-DDE	60	100	0.04	1.73	0.57
o,p′-DDE	0	0	0	0	0
p,p′-DDD	1	1.67	0.01	0.01	0.01
o,p′-DDD	0	0	0	0	0
p,p′-DDT	33	55.0	0.01	0.13	0.02
o,p′-DDT	0	0	0	0	0
**Total DDT**	**60**	**100**	**0.12**	**1.82**	**0.55**
HCB	5	8.33	0.01	0.02	0.01
Sum HCH (α-, β-, γ-)	0	0	0	0	0

Diagnostic ratios are commonly used to assess the age and origin of DDT residues, such as the metabolic ratio (p,p′-DDE + p,p′-DDD)/p,p′-DDT and the isomeric ratio o,p′-DDT/p,p′-DDT. The metabolic ratio had a median value of 24.12 (range 6.93–110.14; *n *= 33), indicating predominance of aged or weathered DDT residues. p,p′-DDD was quantified in only one of the 60 eggs, while o,p′-DDT was below the reporting level in all samples; consequently, the o,p′-DDT/p,p′-DDT isomeric ratio could not be meaningfully evaluated for source attribution. p,p′-DDT was present in 33 out of 60 eggs (55%). Although the presence of parent p,p′-DDT indicates that some undegraded DDT remains in the system, it does not by itself demonstrate recent DDT input, as residual parent DDT may persist in environmental reservoirs and be remobilized into aquatic food webs. The near-exclusive presence of p,p′-DDE, the primary aerobic metabolite of p,p′-DDT, further supports the dominance of legacy sources.

An exploratory two-way linear model identified with Island and Year as factors found no significant effect of either factor or their interaction on contaminant concentrations (Island: *F* (1,56) = 0.074, *p *= 0.786, partial *η*^2^ = 0.001; Year: *F* (1,56) = 1.796, *p *= 0.186, partial *η*^2^ = 0.031; Island × Year interaction: *F* (1,56) = 0.041, *p *= 0.840, partial *η*^2^ = 0.001). However, given the limited 2-year timeframe and modest sample sizes, our study is not designed to detect subtle or long-term temporal trends.

HCB was quantified in a small subset of eggs (mean 0.01 mg/kg, Table 1), while HCH (α-, β-, γ-HCH) was not quantified in any sample. The egg-matrix blank showed calculated concentrations below the reporting level for the HCH isomers and HCB. The absence of HCH indicates that concentrations were below the applied reporting level of 0.01 mg/kg, rather than confirming their complete absence. Similarly, the low frequency of HCB quantification should be interpreted in light of this reporting level. While HCH was not analysed in Holz and Starke (1990), HCB concentrations exceeding more than 0.03 mg/kg (0.03 ppm) were reported for the island BOE in 1986.

DDT and its metabolites as well as PCBs were present in 100% of eggs across all Year-Island groups. Mean (median) of total DDT concentrations were 0.42 (0.37), 0.53 (0.32), 0.63 (0.39), and 0.61 (0.47) mg/kg for 2023-BOE, 2023-RW, 2024-BOE, and 2024-RW, respectively. In contrast, the mean (median) of all PCB congener concentrations was 0.135 (0.099), 0.150 (0.122), 0.167 (0.098), and 0.268 (0.194) mg/kg for the same groups across all samples. DDT and its metabolites were consistently present and markedly lower than PCB concentrations at both islands in both years.

### Contaminant impact on egg parameters

**Fig. 4:**
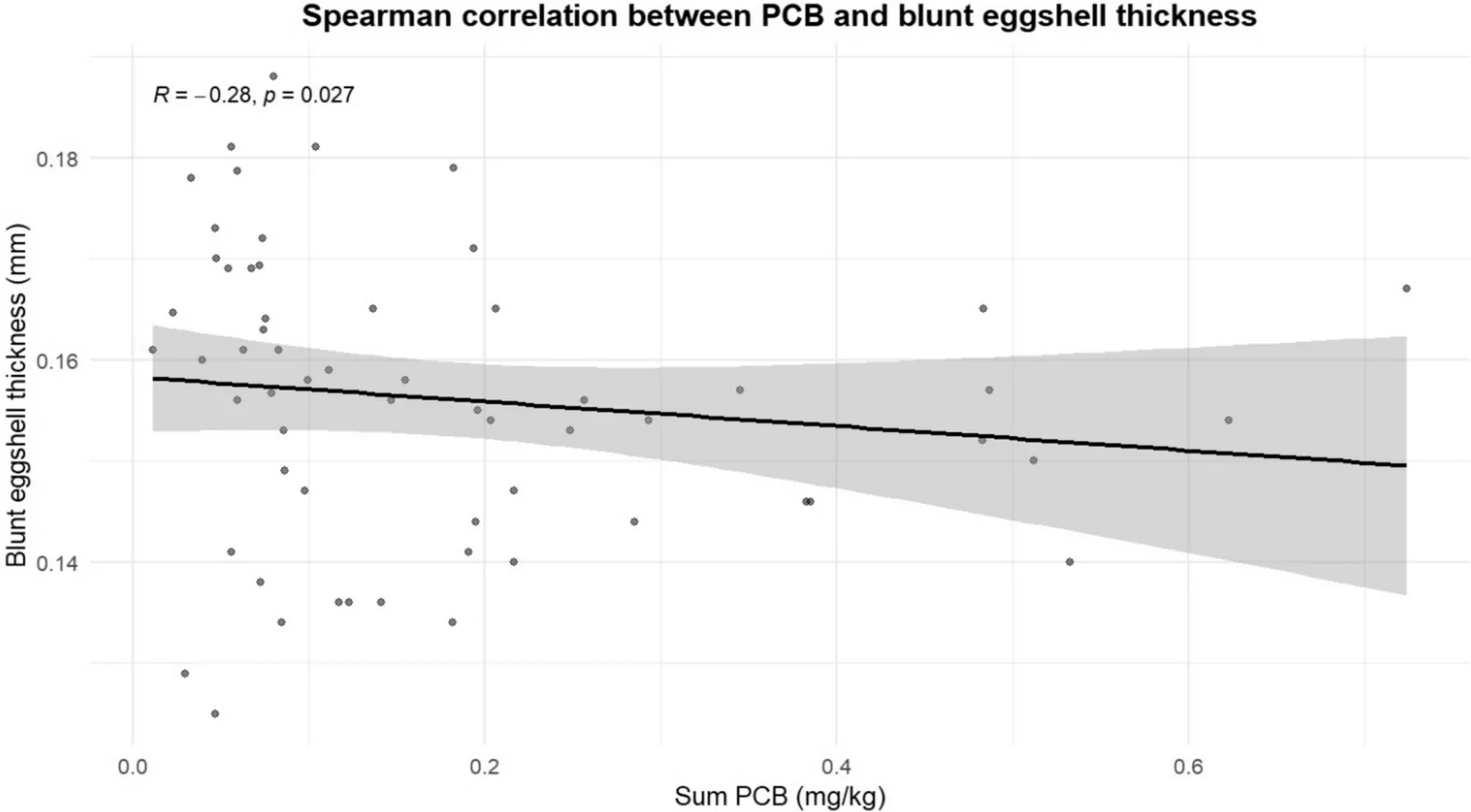
Spearman’s rank correlations between total PCB concentrations and eggshell measurements from the blunt pole. Solid lines represent linear fits with 95% confidence intervals for visualization purposes. Correlation strength and significance were assessed using Spearman’s *ρ* due to non-normal data distributions and the presence of values near reporting levels

No associations were detected between contaminants and eggshell thickness metrics. Spearman’s correlations between all DDT and its metabolites or total PCB and each eggshell metric (blunt, sharp, lateral, average) were small in magnitude and non-significant (all *p *> 0.05, Fig. 4, Supplementary Material 5), except for a negative correlation between the eggshell measurements from the blunt pole and the sum of PCB congeners (*R *= −0.28, *p *= 0.027); the data are providing no evidence that either contaminant influenced eggshell thickness.

Spearman’s rank correlations between egg volume, PCBs, DDT, and its metabolites were consistently low and non-significant (all *p *> 0.05), indicating that egg volume was unrelated to contaminant burden in this dataset.

### Contaminant impact on body parameters of the egg-laying females

Female BMI showed no association with DDT and its metabolites (*p *> 0.05) or PCBs (*p *> 0.05) in Spearman’s correlations. Given the reliance on non-invasive sampling, our study can only infer potential impacts on female physiological condition indirectly, based on biometric data and egg parameters, rather than direct physiological measurement.

## Discussion

Our results show a clear predominance of DDE and a detection frequency of p,p′-DDT of 55% in Black-headed Gull eggs, indicating a dominant legacy signal but also the potential for low-level ongoing input or remobilization. The DDE dominance reflects long-term aerobic transformation and persistent bioaccumulation through the food chain (Qiu & Zhu, 2010; Ricking & Schwarzbauer, 2012; Zhou et al., 2025). In samples where p,p′-DDT was quantified, diagnostic ratio (p,p′-DDE + p,p′-DDD)/p,p′-DDT could be calculated and was consistently low, supporting the conclusion that most contamination reflects aged residues. However, the presence of p,p′-DDT in over half the samples requires careful interpretation regarding potential recent inputs. Furthermore, the absence of quantifiable o,p′-DDE (i.e. concentrations below the reporting level of 0.01 mg/kg), together with the consistently higher concentrations of p,p′-DDE, is consistent with a predominantly technical DDT source (Ricking & Schwarzbauer, 2012). The residue profile of DDT and its metabolites in our samples points to legacy contamination from historical use of technical DDT (the commercial mixture of DDT isomers), but we cannot exclude the possibility of ongoing or remobilized input.

Ecologically, the DDE dominance is relevant given its well-documented association with reproductive effects in birds, such as eggshell thinning (Heath et al., 1969; Hickey & Anderson, 1968; Lundholm, 1997). While eggshell thinning has historically been linked to DDT metabolites, shell thickness alone is not a direct diagnostic tool, as multiple factors can influence this parameter. Our dataset contained minimal DDD, suggesting limited anaerobic conditions in contaminant sources and transport pathways. Despite persistent DDE presence, concentrations in Black-headed Gull eggs remained below published thresholds for eggshell thinning in Herring Gulls (*Larus argentatus*) with concentrations above 160 mg/kg (Becker et al., 1985; Pearce et al., 1979), and no correlations were observed between contaminant concentrations and biometric or morphometric parameters. These findings confirm that acute toxic effects have not been found in the current population, though sublethal impacts cannot be ruled out and should be further monitored.

Comparison with historical data shows remarkable stability in DDE concentrations with reports of Holz and Starke (1990) showing mean DDE concentrations of 0.59 mg/kg in Black-headed Gull eggs from the same region, while our current mean DDE (o,p′ + p,p′) is 0.57 mg/kg. Similar and higher levels have been observed in Black-headed Gull eggs from the North Sea between 0.1 and 1 mg/kg (Becker et al., 1985). Comparisons with historical data should be interpreted with caution due to potential differences in sample type, sample matrix, analytical approaches, and reporting levels. Environmental degradation rates for DDT and its metabolites vary with conditions, with reported half-lives ranging from 2 to 15 years for DDT, 5 to 10 years for DDD, and 10 years or more for DDE (Jayaraj et al., 2016). Dilution of environmental reservoirs may also contribute to the observed trends. The DDT composition in our eggs, dominated by DDE, supports a scenario of aged, oxidized residues and minimal ongoing reduction, indicating limited recent DDT input. A slight decline in DDE since 1990 aligns with regional mussel data from the Baltic Sea (Potrykus et al., 2003), suggesting gradual reduction of DDT legacy in the ecosystem. Available data indicate ongoing exposure in other tissues and regions. For example, in Sweden, Black-headed Gull pectoral muscle samples contained mean DDT concentrations of 0.31 mg/kg (Lindberg et al., 1985), and in Italy, DDT concentrations in liver reached 0.63 mg/kg (Naso et al., 2003). While direct comparisons between tissue types and eggs are limited by differences in accumulation and metabolism, these findings support the conclusion that legacy DDT contamination persists in Black-headed Gulls across Europe.

In comparison to Common Terns (*Sterna hirundo*) or Herring Gulls, the eggs of Black-headed Gulls are more susceptible to DDT contamination (Becker et al., 1985). Notably, Black-headed Gulls, due to their smaller size, may be more sensitive to DDT and PCB-induced reproductive effects than larger gulls such as the Herring Gull (Becker et al., 1985; Lundholm, 1997). This increased sensitivity should be considered when interpreting contaminant thresholds and ecological risks. Generally, when comparing species, caution is warranted. As highlighted by Holz and Starke (1990), Herring Gulls are more frequently studied than Black-headed Gulls, and direct comparisons between these species are complicated unless data collection is conducted concurrently and based on the same principles. Nevertheless, ongoing monitoring is essential given DDT’s persistence, bioaccumulation potential, spatial variability, and possible remobilization of DDT residues.

Our PCB results show a profile dominated by higher-chlorinated congeners (PCB-138, PCB-153, PCB-180), consistent with persistence and bioaccumulation patterns in aquatic birds. Lighter congeners were detected less frequently, supporting a classic legacy PCB profile. Heavier PCBs have low water solubility and bind preferentially to organic-rich particles, whereas lighter congeners remain more dissolved (Zhou et al., 2025). Holz and Starke (1990) reported total PCBs, not individual congeners, in Black-headed Gull eggs from the same region. To allow valid comparison, we summed the six target PCB congeners in our present dataset. Our total mean PCB (0.18 mg/kg) is substantially lower than the > 2 mg/kg reported in 1986, indicating a pronounced decline in PCB burden over the past four decades. Historical data from Herring Gull eggs in the North Sea show similar dominance of higher-chlorinated PCBs and declining levels through the 1990 s (Fliedner et al., 2012). Effects are suspected at PCB concentrations of 3–5 mg/kg in Herring Gulls, although similar PCB levels have been reported in the smaller Black-headed Gull (Becker et al., 1985). In comparison, *Mytilus trossulus* (blue mussel) tissue from the Pomeranian Bay near Riether Werder Island shows much lower PCB concentrations (Potrykus et al., 2003), even though PCBs biomagnify in gulls’ opportunistic diet of small fish, insects, and molluscs.

HCB was quantified in only five samples from Riether Werder, near the Polish border, and was not quantifiable on Boehmke Island, near Usedom. It is possible that low-level HCB quantifications reflect diffuse background contamination; however, targeted source analysis was beyond the scope of this study. Earlier studies reported no HCB in perch from the Szczecin Lagoon (Falandysz et al., 2004) and much higher HCB levels in Black-headed Gull eggs from Usedom in the 1990 s (Holz & Starke, 1990). Low-level HCB was present in a small subset of eggs, but no conclusion about site-specific sources can be drawn without additional data. HCH was not quantified at either site, aligning with the rapid biotic degradation (Sagunski & Perger, 1994) and suggesting no substantial recent inputs. While Black-headed Gull eggs from the Elbe estuary previously showed high γ-HCH (Lindane) concentrations (Becker et al., 1985), our results do not indicate comparable contamination in the recent Baltic Sea samples.

Egg morphometrics and female BMI were stable across islands and years, further supporting the lack of detectable contaminant impact on reproductive parameters. In wild birds, elevated DDT levels often co-occur with other pollutants such as PCBs, making it difficult to isolate effects of individual compounds (Lundholm, 1997). The limited HCB presence, no quantifiable levels of HCHs, and predominance of DDT and its metabolites across time and space point to legacy contamination with little evidence for recent or weather-driven inputs. Persistent pollutants in avian eggs demonstrate continued exposure and maternal transfer, with potential for sublethal effects on reproduction, development, and population viability. However, the sample size and detection frequencies limit statistical power, so non-significant results should be interpreted with caution. It should be noted that the 2 study years represent only a small-time window which should be further monitored. Additionally, only indirect measures of female physiological condition were available, as we relied on biometric data and egg characteristics from one sampling event. Therefore, our conclusions regarding maternal health and contaminant effects are necessarily inferential. A key limitation of our study is that eggs were collected at random without determining their laying order. As previous research shows that the first-laid egg in a clutch often contains higher concentrations of persistent organic pollutants (Li et al., 2021; Van Den Steen et al., 2009; Verreault et al., 2006), our sampling approach may introduce additional variance and potential bias. Future studies should record and control for laying order to minimize this source of bias.

Historical data interpretation is limited by incomplete methodological details and compound specification (Holz & Starke, 1990). Broader Baltic Sea studies show a sevenfold reduction in PCBs, HCBs, DDT, and its metabolites’ concentrations since the 1980 s (Zhou et al., 2025). Despite positive trends, legacy pollutants remain present in remote areas.

Our results provide essential first data for ongoing biomonitoring programmes in this region. Such data are crucial for tracking temporal trends, identifying emerging risks, and informing future assessments of contaminant exposure in avian bioindicators. Future work combining hydrodynamic data and compound-specific source apportionment could further elucidate transport pathways and the roles of local versus diffuse legacy reservoirs. The quantification of DDT and PCB residues at these levels, decades after their ban, highlights the ongoing transfer through the food web, the persistence of these compounds and the need for ongoing surveillance in the Baltic Sea coastal ecosystem.

## Conclusion

This study demonstrates that legacy pollutants, including PCBs and DDT metabolites, remain widely present in Black-headed Gull eggs from the southern Baltic Sea, despite regulatory bans decades ago. While PCB concentrations appear to have declined substantially since the 1980 s, DDE concentrations are similar to those reported in the 1990 s, though differences in analytical methods and sample composition limit direct comparison. The absence of statistically significant associations between contaminant concentrations and biometric parameters in this dataset does not necessarily indicate uniform exposure or accumulation patterns and may reflect limited sample size or unmeasured variables. Continued monitoring is essential to track long-term trends, evaluate potential remobilization from environmental reservoirs, and provide early warning of renewed exposure or unexpected ecological effects. By providing updated baseline data for a previously understudied species in this region, our findings reinforce the need for surveillance of even well-studied, regulated substances like DDT to ensure the effectiveness of management measures and to safeguard environmental and wildlife health. This study establishes a critical reference point for future monitoring and highlights the importance of Black-headed Gulls as effective bioindicators of persistent chemical threats in coastal environments.

## Supplementary Information

Below is the link to the electronic supplementary material.

ESM 1 (DOCX 46.8 KB)

## Data Availability

All data supporting the findings of this study are available within the paper and its Supplementary Information.

## References

[CR1] Baker, J. (1993). *Identification of European non-passerines. A BTO Guide* *24*.

[CR2] Becker, P. H., Büthe, A., & Heidmann, W. (1985). Schadstoffe in Gelegen von Brutvögeln der deutschen Nordseeküste. I. Chlororganische Verbindungen. *Journal Für Ornithologie,**126*(1), 29–51. 10.1007/BF01640441

[CR3] Becker, P. H., Heidmann, W. A., Büthe, A., Frank, D., & Koepff, C. (1992). Umweltchemikalien in Eiern von Brutvögeln der deutschen Nordseeküste: Trends 1981–1990. *Journal Für Ornithologie,**133*(2), 109–124. 10.1007/BF01639904

[CR4] Ben-Shachar, M., Lüdecke, D., & Makowski, D. (2020). Effectsize: Estimation of effect size indices and standardized parameters. *Journal of Open Source Software,**5*(56), Article 2815. 10.21105/joss.02815

[CR6] Beyer, A., & Biziuk, M. (2009). Environmental fate and global distribution of polychlorinated biphenyls. In D. M. Whitacre (Ed.), *Reviews of environmental contamination and toxicology vol 201* (Vol. Vol. 201, pp. 137–158). Springer US. 10.1007/978-1-4419-0032-6_5 19484591

[CR7] Dabrowska, H., Kopko, O., Lehtonen, K. K., Lang, T., Waszak, I., Balode, M., & Strode, E. (2017). An integrated assessment of pollution and biological effects in flounder, mussels and sediment in the southern Baltic Sea coastal area. *Environmental Science and Pollution Research,**24*(4), 3626–3639. 10.1007/s11356-016-8117-8 27885579

[CR8] Dahlberg, A.-K., Apler, A., Vogel, L., Wiberg, K., & Josefsson, S. (2020). Persistent organic pollutants in wood fiber–contaminated sediments from the Baltic Sea. *Journal of Soils and Sediments,**20*(5), 2471–2483. 10.1007/s11368-020-02610-6

[CR9] Daley, J. M., Corkum, L. D., & Drouillard, K. G. (2011). Aquatic to terrestrial transfer of sediment associated persistent organic pollutants is enhanced by bioamplification processes. *Environmental Toxicology and Chemistry,**30*(9), 2167–2174. 10.1002/etc.608 21713972

[CR10] European Commission. (2026). Analytical quality control and method validation procedures for pesticide residues analysis in food and feed. SANTE/11312/2021. https://www.eurl-pesticides.eu/userfiles/file/EurlALL/SANTE-11312_2021-V2026.pdf. Accessed 10 June 2026

[CR11] Falandysz, J., Wyrzykowska, B., Warzocha, J., Barska, I., Garbacik-Wesołowska, A., & Szefer, P. (2004). Organochlorine pesticides and PCBs in perch *Perca fluviatilis* from the Odra/Oder river estuary, Baltic Sea. *Food Chemistry,**87*(1), 17–23. 10.1016/j.foodchem.2003.10.011

[CR12] Fenstad, A. A., Jenssen, B. M., Gabrielsen, K. M., Öst, M., Jaatinen, K., Bustnes, J. O., et al. (2016). Persistent organic pollutant levels and the importance of source proximity in Baltic and Svalbard breeding common eiders. *Environmental Toxicology and Chemistry,**35*(6), 1526–1533. 10.1002/etc.3303 26553455

[CR13] Fliedner, A., Rüdel, H., Jürling, H., Müller, J., Neugebauer, F., & Schröter-Kermani, C. (2012). Levels and trends of industrial chemicals (PCBs, PFCs, PBDEs) in archived herring gull eggs from German coastal regions. *Environmental Sciences Europe,**24*(1), 7. 10.1186/2190-4715-24-7

[CR14] Fox, J., & Weisberg, S. (2024). *Car: An R companion to applied regression* (3rd ed.). Sage.

[CR15] Friedland, R., Schernewski, G., Gräwe, U., Greipsland, I., Palazzo, D., & Pastuszak, M. (2019). Managing eutrophication in the Szczecin (Oder) lagoon-development, present state and future perspectives. *Frontiers in Marine Science,**5*, 521. 10.3389/fmars.2018.00521

[CR16] Garnier, S., Ross, N., BoB Rudis, Filipovic-Pierucci, A., Galili, T., Timelyportfolio, et al. (2023). sjmgarnier/viridis: CRAN release v0.6.3. Zenodo. 10.5281/ZENODO.4679423

[CR17] Heath, R. G., Spann, J. W., & Kreitzer, J. F. (1969). Marked DDE impairment of mallard reproduction in controlled studies. *Nature,**224*(5214), 47–48. 10.1038/224047a0 5822906

[CR18] Herrmann, C. (2017). Jahresbericht der AG Küstenvogelschutz Mecklenburg-Vorpommern 2017: Aktivitäten der AG Küstenvogelschutz und Brutergebnisse in den Küstenvogelbrutgebieten Mecklenburg-Vorpommerns. *Seevögel,**39*(4), 10–17.

[CR19] Herrmann, C., Schröder, C., Schmitz-Ornés, A., Seifert, N., & Olsthoorn, G. (2022). Jahresbericht der AG Küstenvogelschutz Mecklenburg-Vorpommern 2021: Aktivitäten und Brutergebnisse in den Küstenvogelbrutgebieten. *Seevögel,**44*(3–4), 25–32.

[CR20] Hickey, J. J., & Anderson, D. W. (1968). Chlorinated hydrocarbons and eggshell changes in raptorial and fish-eating birds. *Science,**162*(3850), 271–273. 10.1126/science.162.3850.271 4877438

[CR21] Hildmann, F., Gottert, C., Frenzel, T., Kempe, G., & Speer, K. (2015). Pesticide residues in chicken eggs – A sample preparation methodology for analysis by gas and liquid chromatography/tandem mass spectrometry. *Journal of Chromatography A,**1403*, 1–20. 10.1016/j.chroma.2015.05.024 26051081

[CR22] Holz, R., & Starke, W. (1984). Biometrie, Geschlechtsdimorphismus und Eimaße mecklenburgischer Lachmöwen (*Larus ridibundus*)*: Beitr. Vogelkd., Jena 30 , *4/5. Jena.

[CR23] Holz, R., & Starke, W. (1990). Fremdstoffbelastung in Eiern mecklenburgischer Lachmöwen (*Larus ridibundus*) *von 1975 bis 1986: Arch. Nat.schutz Landsch.forsch., Berlin 30 (1990) 1*. Berlin: Arch. Nat.schutz Landsch.forsch. https://sammlungen.ub.uni-frankfurt.de/biodiv/periodical/titleinfo/12712644. Accessed 30 May 2026

[CR24] Indykiewicz, P., Manuta, N., Bonecka, J., Gündemir, O., & Szara, T. (2025). Is the skull of the black-headed gull dimorphic? Analysis of shape variation. *The European Zoological Journal,**92*(1), 174–181. 10.1080/24750263.2025.2450460

[CR25] Jayaraj, R., Megha, P., & Sreedev, P. (2016). Review article. Organochlorine pesticides, their toxic effects on living organisms and their fate in the environment. *Interdisciplinary Toxicology,**9*(3–4), 90–100. 10.1515/intox-2016-0012 PMC5464684 28652852

[CR26] Jonsson, P., Eckhéll, J., & Larsson, P. (2000). PCB and DDT in laminated sediments from offshore and archipelago areas of the NW Baltic Sea. *Ambio: A Journal of the Human Environment,**29*(4), 268–276. 10.1579/0044-7447-29.4.268

[CR27] Kannan, K., Corsolini, S., Falandysz, J., Oehme, G., Focardi, S., & Giesy, J. P. (2002). Perfluorooctanesulfonate and related fluorinated hydrocarbons in marine mammals, fishes, and birds from coasts of the Baltic and the Mediterranean Seas. *Environmental Science & Technology,**36*(15), 3210–3216. 10.1021/es020519q 12188342

[CR28] Knudtzon, N. C., Thorstensen, H., Ruus, A., Helberg, M., Bæk, K., Enge, E. K., & Borgå, K. (2021). Maternal transfer and occurrence of siloxanes, chlorinated paraffins, metals, PFAS and legacy POPs in herring gulls (*Larus argentatus*) of different urban influence. *Environment International,**152*, Article 106478. 10.1016/j.envint.2021.106478 33770583

[CR29] Kumari, K., Shahbaz, M., & Raghunathan, K. (2024). Hexachlorocyclohexane (α, β-HCH) and lindane (γ-HCH). In K. Kumari (Ed.), *Pollutants of global concern: A comprehensive overview of persistent organic pollutants* (pp. 165–180). Springer International Publishing. 10.1007/978-3-031-50996-4_12

[CR30] Kuo, D. T. F., Rattner, B. A., Marteinson, S. C., Letcher, R., Fernie, K. J., Treu, G., et al. (2022). A critical review of bioaccumulation and biotransformation of organic chemicals in birds. *Reviews of Environmental Contamination and Toxicology,**260*(1), 6. 10.1007/s44169-021-00007-1

[CR31] Li, Z.-R., Luo, X.-J., Lin, L., Zeng, Y.-H., & Mai, B.-X. (2021). Effect of laying sequence and selection of maternal tissues in assessment of maternal transfer of organohalogenated contaminants during chicken egg formation: A pilot study. *Environmental Pollution,**270*, Article 116157. 10.1016/j.envpol.2020.116157 33321435

[CR32] Lindberg, P., Odsjö, T., & Reutergårdh, L. (1985). Residue levels of polychlorobiphenyls, ΣDDT, and mercury in bird species commonly preyed upon by the peregrine falcon (Falco peregrinus Tunst.) in Sweden. *Archives of Environmental Contamination and Toxicology*, *14*(2), 203–212. 10.1007/BF01055613 3920979

[CR33] Lüdecke, D. (2017). ggeffects: Create tidy data frames of marginal effects for “ggplot” from model outputs. 10.32614/CRAN.package.ggeffects

[CR34] Lundholm, C. E. (1997). DDE-induced eggshell thinning in birds: Effects of p,p′-DDE on the calcium and prostaglandin metabolism of the eggshell gland. *Comparative Biochemistry and Physiology Part C: Pharmacology, Toxicology and Endocrinology,**118*(2), 113–128. 10.1016/S0742-8413(97)00105-9 9490182

[CR35] Makatsch, W. (1952). *Die Lachmöwe* (1st ed.). A. Ziemsen Verlag.

[CR36] Naso, B., Perrone, D., Ferrante, M. C., Zaccaroni, A., & Lucisano, A. (2003). Persistent organochlorine pollutants in liver of birds of different trophic levels from coastal areas of Campania, Italy. *Archives of Environmental Contamination and Toxicology,**45*(3), 407–414. 10.1007/s00244-003-2201-z 14674594

[CR37] Neumann, T., Schernewski, G., & Friedland, R. (2025). Transformation processes in the Oder Lagoon as seen from a model perspective. *Biogeosciences,**22*(22), 7403–7424. 10.5194/bg-22-7403-2025

[CR38] Ornés, A. S., Herbst, A., Spillner, A., Mewes, W., & Rauch, M. (2014). A standardized method for quantifying eggshell spot patterns: Method for measuring eggshell spot patterns. *Journal of Field Ornithology,**85*(4), 397–407. 10.1111/jofo.12079

[CR39] Padayachee, K., Reynolds, C., Mateo, R., & Amar, A. (2023). A global review of the temporal and spatial patterns of DDT and dieldrin monitoring in raptors. *Science of the Total Environment,**858*, Article 159734. 10.1016/j.scitotenv.2022.159734 36349626

[CR40] Pazikowska-Sapota, G. (2006). Persistent organic pollutants (POPs) in bottom sediments from the Baltic Sea. *Oceanological and Hydrobiological Studies*, *35*.

[CR41] Pearce, P. A., Peakall, D. B., & Reynolds, L. M. (1979). Shell thinning and residues of organochlorines and mercury in seabird eggs, Eastern Canada, 1970–76. *Pesticides Monitoring Journal,**13*(2), 61–68. 117427

[CR42] Peig, J., & Green, A. J. (2009). New perspectives for estimating body condition from mass/length data: The scaled mass index as an alternative method. *Oikos,**118*(12), 1883–1891. 10.1111/j.1600-0706.

[CR43] Peterson, S. H., Ackerman, J. T., Herzog, M. P., Toney, M. S., Cooney, B., & Hartman, C. A. (2020). Avian eggshell thickness in relation to egg morphometrics, embryonic development, and mercury contamination. *Ecology and Evolution,**10*(16), 8715–8740. 10.1002/ece3.6570 PMC7452760 32884653

[CR44] Petrlik, J., Bell, L., DiGangi, J., Allo’o Allo’o, S. M., Kuepouo, G., Ochola, G. O., et al. (2022). Monitoring dioxins and PCBs in eggs as sensitive indicators for environmental pollution and global contaminated sites and recommendations for reducing and controlling releases and exposure. *Emerging Contaminants*, *8*, 254–279. 10.1016/j.emcon.2022.05.001

[CR45] Piotrowski, S. (2003). Presence of polychlorinated biphenyls (PCBs) and organochlorine pesticides (OCPs) in bottom sediments of the Odra River estuary (NW Poland). *Przegląd Geologiczny,**51*(10), 835–840.

[CR46] Potrykus, J., Albalat, A., Pempkowiak, J., & Porte, C. (2003). Content and pattern of organic pollutants (PAHs, PCBs and DDT) in blue mussels *(Mytilus trossulus)* from the southern Baltic Sea. *Oceanologia,**45*(2), 337–355.

[CR47] Price, E. R. (2017). The physiology of lipid storage and use in reptiles. *Biological Reviews,**92*(3), 1406–1426. 10.1111/brv.12288 27348513

[CR48] Pusch, K., Schlabach, M., Prinzinger, R., & Wing Gabrielsen, G. (2005). Gull eggs—Food of high organic pollutant content? *Journal of Environmental Monitoring,**7*(6), 635–639. 10.1039/B501879J 15931427

[CR49] Qiu, X., & Zhu, T. (2010). Using the o,p′-DDT/p,p′-DDT ratio to identify DDT sources in China. *Chemosphere,**81*(8), 1033–1038. 10.1016/j.chemosphere.2010.08.049 20851452

[CR50] Ratcliffe, D. (1967). Decrease in eggshell weight in certain birds of prey. *Nature,**215*(5097), 208–210. 10.1038/215208a0 6049131

[CR51] Reckermann, M., Omstedt, A., Soomere, T., Aigars, J., Akhtar, N., Bełdowska, M., et al. (2022). Human impacts and their interactions in the Baltic Sea region. Earth System Dynamics. 10.5194/esd-13-1-2022

[CR52] Reindl, A., Falkowska, L., & Grajewska, A. (2019). Halogenated organic compounds in the eggs of aquatic birds from the Gulf of Gdansk and Wloclawek Dam (Poland). *Chemosphere,**237*, Article 124463. 10.1016/j.chemosphere.2019.124463 31382198

[CR53] Reindl, A., Falkowska, L., & Grajewska, A. (2020). Hexabromocyclododecane contamination of herring gulls in the coastal area of the southern Baltic Sea. *Oceanological and Hydrobiological Studies,**49*(2), 147–156. 10.1515/ohs-2020-0014

[CR54] Ricking, M., & Schwarzbauer, J. (2012). DDT isomers and metabolites in the environment: An overview. *Environmental Chemistry Letters,**10*(4), 317–323. 10.1007/s10311-012-0358-2

[CR55] Robinson, D., Hayes, A., & Couch, S. (2014). broom: Convert statistical objects into tidy tibbles. 10.32614/CRAN.package.broom

[CR56] Ryslavy, T., Bauer, H. G., Gerlach, B., Hüppop, O., Stahmer, J., Südbeck, P., & Sudfeldt, C. (2020). Rote Liste der Brutvögel Deutschlands, 6. Fassung, 30. September 2020. *Berichte Zum Vogelschutz,**57*, 13–112.

[CR57] Sagunski, H., & Perger, G. (1994). *Biozide. In: Lehrbuch der Toxikologie*. Mannheim: BI Wissenschaftsverlag.

[CR58] Sánchez, J., Solé, M., & Albaigés, J. (1993). A comparison of distributions of PCB congeners and other chlorinated compounds in fishes from coastal areas and remote lakes. *International Journal of Environmental Analytical Chemistry,**50*(4), 269–284. 10.1080/03067319308027603

[CR59] Sebastiano, M., Jouanneau, W., Blévin, P., Angelier, F., Parenteau, C., Gernigon, J., et al. (2021). High levels of fluoroalkyl substances and potential disruption of thyroid hormones in three gull species from South Western France. *Science of the Total Environment,**765*, Article 144611. 10.1016/j.scitotenv.2020.144611 33385816

[CR60] Sebastiano, M., Jouanneau, W., Blévin, P., Angelier, F., Parenteau, C., Pallud, M., et al. (2023). Physiological effects of PFAS exposure in seabird chicks: A multi-species study of thyroid hormone triiodothyronine, body condition and telomere length in South Western France. *Science of the Total Environment,**901*, Article 165920. 10.1016/j.scitotenv.2023.165920 37527721

[CR61] Sun, C. J., Chen, S. R., Xu, G. Y., Liu, X. M., & Yang, N. (2012). Global variation and uniformity of eggshell thickness for chicken eggs. *Poultry Science,**91*(10), 2718–2721. 10.3382/ps.2012-02220 22991562

[CR62] Szlinder-Richert, J., Usydus, Z., & Drgas, A. (2012). Persistent organic pollutants in sediment from the southern Baltic: Risk assessment. *Journal of Environmental Monitoring,**14*(8), 2100–2107. 10.1039/C2EM30221G 22751780

[CR63] Thorstensen, H., Ruus, A., Helberg, M., Bæk, K., Enge, E. K., & Borgå, K. (2021). Common eider and herring gull as contaminant indicators of different ecological niches of an urban fjord system. *Integrated Environmental Assessment and Management,**17*(2), 422–433. 10.1002/ieam.4340 32926521

[CR64] Troscianko, J. (2014). A simple tool for calculating egg shape, volume and surface area from digital images. *Ibis,**156*(4), 874–878. 10.1111/ibi.12177

[CR65] Troscianko, J, & Stevens, M. (2019). micaToolbox user guide (version 2.2). https://www.empiricalimaging.com. Accessed 15 July 2025

[CR66] Van Den Steen, E., Pinxten, R., Jaspers, V. L. B., Covaci, A., Barba, E., Carere, C., et al. (2009). Brominated flame retardants and organochlorines in the European environment using great tit eggs as a biomonitoring tool. *Environment International,**35*(2), 310–317. 10.1016/j.envint.2008.08.002 18804864

[CR67] Verreault, J., Berger, U., & Gabrielsen, G. W. (2007). Trends of perfluorinated alkyl substances in herring gull eggs from two coastal colonies in northern Norway: 1983–2003. *Environmental Science & Technology,**41*(19), 6671–6677. 10.1021/es070723j 17969679

[CR68] Verreault, J., Villa, R. A., Gabrielsen, G. W., Skaare, J. U., & Letcher, R. J. (2006). Maternal transfer of organohalogen contaminants and metabolites to eggs of Arctic-breeding glaucous gulls. *Environmental Pollution,**144*(3), 1053–1060. 10.1016/j.envpol.2005.10.055 16563578

[CR69] Wickham, H. (2016). *Ggplot2: Elegant graphics for data analysis*. Springer-Verlag.

[CR70] Wickham, H., Averick, M., Bryan, J., Chang, W., McGowan, L., François, R., et al. (2019). Welcome to the tidyverse. *Journal of Open Source Software,**4*(43), Article 1686. 10.21105/joss.01686

[CR71] Wickham, H., & Bryan, J. (2025). readxl: Read Excel files. https://readxl.tidyverse.org

[CR72] Wickham, H., François, R., Henry, L., Müller, K., & Vaughan, D. (2023). dplyr: A grammar of data manipulation. https://CRAN.R-project.org/package=dplyr

[CR73] Wickham, H., & Henry, L. (2025). purrr: Functional programming tools. https://purrr.tidyverse.org/

[CR74] Zhou, M., Hand, I., Lerz, A., Moros, M., Schulz Bull, D. E., & Waniek, J. J. (2025). Temporal development of chlorinated hydrocarbons in the Baltic Sea sediments: Characterization of the pollution maximum. *Science of the Total Environment,**962*, Article 178395. 10.1016/j.scitotenv.2025.178395 39799649

[CR75] Zhu, H. (2017). kableExtra: Construct complex table with “kable” and pipe syntax. 10.32614/CRAN.package.kableExtra

