# Supplementary material for: Still exposed? DDT and PCB levels in Black-headed Gull eggs collected from the Baltic Sea
Source: Environ Monit Assess. 2026 Oct 7;198(11):1156. doi: 10.1007/s10661-026-15987-7 (PMC13645991; doi:10.1007/s10661-026-15987-7)
Supplement: Supplementary file 1 [file 10661_2026_15987_MOESM1_ESM.docx]

# Supplementary Material

## Supplementary Material 1. Materials and reagents

If not further specified, chemicals had analytical purity between 98 – 99%, water had appropriate purity, and all organic solvents have met the requirements for residue analysis. The term ‘solution’ refers to an aqueous solution. Stock solutions are prepared from solids (with certificate, e.g. from LGC / Ehrenstorfer, Sigma Aldrich and Restek). Due to the number and different chemical properties of the active substances to be determined, we prepared several standard mixtures of the pesticides. A concentration of 10 ng/μL is required for preparing the calibration solutions and the internal standard solution.

The following reagents have been used:

1. Cyclohexane, e.g. p.A. from Promochem

2. Hexane, e.g. p.A. from Promochem

3. Ethyl acetate (EEE), e.g. p.A. from Promochem

4. Isooctane, e.g. suprasolv from Merck

5. Acetone, e.g. p.A. from Merck

6. Extraction mixture: hexane/acetone mixture, 2:1

7. GPC elution mixture: ethyl acetate/cyclohexane mixture, 1:1

8. Sodium sulphate, anhydrous, fine-grained, heated to 550°C for at least 2 hours

9. Cotton wool thoroughly extracted with acetone

## Supplementary Material 2. Analytical quality control and method validation results for target compounds in egg matrix.

| **Compound** | **Initial recovery (%)** | **Initial RSD (%)** | **n** | **Ongoing recovery (%)** | **Ongoing RSD (%)** | **n** |
| --- | --- | --- | --- | --- | --- | --- |
| α-HCH | 106.8 | 4.4 | 5 | 98.5 | 12.1 | 8 |
| β-HCH | 108.6 | 7.2 | 5 | 105.1 | 17.0 | 7 |
| γ-HCH | 112.8 | 2.2 | 5 | 103.3 | 12.5 | 8 |
| HCB | 110.5 | 4.6 | 5 | 99.7 | 13.6 | 8 |
| p,p’-DDT | 113.0 | 9.15 | 5 | 101.2 | 15.3 | 7 |
| o,p’-DDT | 123.1 | 5.2 | 5 | 102.9 | 17.1 | 7 |
| p,p’-DDE | 132.3 | 2.7 | 5 | 95.5 | 18.4 | 7 |
| o,p’-DDE | 120.0 | 7.2 | 5 | 100.6 | 17.7 | 6 |
| p,p’-DDD | 124.8 | 5.4 | 5 | 102.9 | 14.3 | 7 |
| o,p’-DDD | 121.7 | 5.4 | 5 | 109.7 | 14.8 | 8 |
| PCB 28 | 117.5 | 5.5 | 5 | 95.1 | 11.0 | 4 |
| PCB 52 | 117.2 | 2.9 | 5 | 95.8 | 17.6 | 4 |
| PCB 138 | 107.3 | 20.7 | 5 | 98.9 | 7.4 | 4 |
| PCB 153 | 126.7 | 5.5 | 5 | 95.1 | 10.1 | 4 |
| PCB 180 | 125.7 | 3.2 | 5 | 95.2 | 8.6 | 4 |

Recovery represents the percentage of the nominal concentration recovered in matrix-matched quality-control samples. RSD = relative standard deviation (RSD, %), n = number of QC measurements included in calculation. Acceptance criteria for initial method validation were 70 – 120% recovery and RSD < 20 %. For routine ongoing QC samples, recoveries between 60 and 140 % were considered acceptable. The lowest successfully validated concentration level was 0.001mg/kg; however, a reporting level (RL) of 0.01mg/kg was applied to the quantitative evaluation of gull egg samples.

Supplementary Material 3. Egg volume in cm^3^ across both study years.

| **Year** | **n** | **Min. (cm^3^)** | **Max. (cm^3^)** | **Mean (cm^3^)** | **SD (cm^3^)** |
| --- | --- | --- | --- | --- | --- |
| 2023 | 27 | 29.72 | 44.78 | 37.05 | 37.99 |
| 2024 | 30 | 28.82 | 46.57 | 36.25 | 37.38 |

Supplementary Material 4. Detection frequencies and concentrations of individual PCB congeners in Black-headed Gull eggs from all years combined (n = 60). Minimum, maximum, and mean concentrations were calculated based on detected samples only. Non-detected concentrations were excluded from these summary statistics.

| **Compounds** | **n detected (absolute)** | **% detected** | **Minimum (mg/kg)** | **Maximum (mg/kg)** | **Mean (mg/kg)** |
| --- | --- | --- | --- | --- | --- |
| PCB-28 | 27 | 45.0 | 0.46 | 7.65 | 1.18 |
| PCB-52 | 7 | 11.7 | 0.61 | 1.22 | 0.89 |
| PCB-101 | 28 | 46.7 | 0.65 | 8.17 | 3.47 |
| PCB-138 | 60 | 100 | 5.83 | 280.14 | 79.27 |
| PCB-153 | 60 | 100 | 3.83 | 308.02 | 61.32 |
| PCB-180 | 60 | 100 | 1.46 | 175.06 | 36.92 |

Supplementary Material 5. Spearman’s rank correlations rho two sided for correlations between each contaminant (DDT and its metabolites, total PCB) and each eggshell metric (blunt, sharp, lateral, average).

| **Predictor** | **Response** | **Estimate** | **p value** |
| --- | --- | --- | --- |
| DDT | Eggshell average | 0.192 | 0.141 |
| DDT | Eggshell sharp | 0.192 | 0.143 |
| DDT | Eggshell blunt | 0.100 | 0.447 |
| DDT | Eggshell lateral | 0.156 | 0.234 |
| DDT | Egg volume | 0.008 | 0.948 |
| PCBs | Eggshell average | 0.212 | 0.105 |
| PCBs | Eggshell sharp | 0.285 | **0.0273** |
| PCBs | Eggshell blunt | 0.0963 | 0.464 |
| PCBs | Eggshell lateral | 0.174 | 0.183 |
| PCBs | Egg volume | 0.125 | 0.353 |
